# Fellow-Workers and Their Doings

**Published:** 1914-04-25

**Authors:** 


### ROUND THE WORLD.

### Fellow-Workers and their Doings.

[The Editor Invites Early Information and Contributions Marked "Fellow-Workers."]

It may be wondered what further scientific honours can fall to the lot of Professor Sherrington, the distinguished occupant of the Chair of Physiology at Liverpool University, who has just been awarded the Actonian prize of the Royal Institution. A fellow of the Royal Society and an honorary LL.D. of five Universities, Dr. Charles Scott Sherrington can also write the letters M.A., M.D., D.Sc. Cantab., M.R.C.S. Eng., F.R.C.P. Lond., after his name, whilst innumerable awards and important lectureships have been his both in this country and abroad. Amongst these may be particularly mentioned the Royal Society's medal and the Baly gold medal of the Royal College of Physicians, and the Charcot medal of the Societé de Névrologie of Paris. Specialising in physiology from early years, Sherrington did not shrink from facing the difficult problems of cerebro-spinal function that had baffled previous workers, and his work on the integrative action of the nervous has placed the whole subject of brain physiology on a sounder scientific basis than it ever had before. Practitioners who passed through St. Thomas's in the days when Sherrington presided over the physiological laboratories there have a vivid recollection of his energy.

Mr. Raymond Johnson, who has been promoted to the senior staff at University College Hospital, qualified from that medical centre as an M.R.C.S. in 1885, three years later taking the F.R.C.S. Mr. Johnson had a most distinguished career as a student, and at the M.B. examination obtained the double distinction and scholarship in forensic medicine, honours in medicine, and gold medal in obstetric medicine. He has, of course, been a member on the staff at University College Hospital and teacher of operative surgery to the medical school for some time, in addition to which he is an examiner at both London and Cambridge Universities. Mr. Raymond Johnson was one of those who went to the front in the South African war, when he was surgeon to the Imperial Yeomanry Hospital; formerly he was on the staffs at the Great Northern Hospital and Victoria Hospital for Children.

Mr. Wilfred Trotter, who succeeds to the senior staff at the same hospital on the resignation of Sir Rickman Godlee, the President of the Royal College of Surgeons, has been assistant surgeon there for some years, and also holds the appointments of surgeon at the East London Hospital, and assistant surgeon at the West End Hospital for Nervous Diseases. Mr. Trotter became a F.R.C.S. in 1899, having qualified M.R.C.S., L.R.C.P. three years previously. He is also an M.D., M.S. Lond., and obtained first-class honours in medicine at the M.B. examination, whilst he won the gold medal and scholarship at the B.S.

Sir Thomas Oliver, M.D. Glasg., F.R.C.P. Lond., whose references to "cancer-houses" in a recent address to the Insurance Institute at Newcastle-on-Tyne have occasioned much interest, is professor of the principles and practice of medicine in Durham University, and served many years on the active staff of the Royal Victoria Infirmary at Newcastle-on-Tyne, to which he is now consulting physician. One of our foremost authorities on industrial diseases, Sir Thomas Oliver is sure of an attentive audience for any pronouncement he may make in regard to public health matters, and his warning as to the dangers of over-athleticism, also dealt with in the aforementioned lecture, will doubtless have a wide influence in moulding the views of family doctors whose advice is sought in this connection.

When Dr. Heaton C. Howard determined to relate his experiences of the potato as a therapeutic agent he, doubtless, little dreamed how much publicity would be achieved by his interesting records. Nevertheless, his recent paper to the *Lancet* (April 11) has been widely commented on, while the *Times* drew attention to it in a special article on Nature's medicines. Dr. Howard is a well-known Stockwell practitioner, who lectures for the St. John Ambulance Association, and an Honorary Associate of the Order of St. John of Jerusalem in England.

Dr. Frank Shufflebotham, M.D. Cantab., who has arranged to begin a course of lectures at Guy's next month dealing with industrial diseases, is one of our best authorities on disorders of health associated with mining and other industries, and lately delivered the Milroy lectures before the Royal College of Physicians. An old Cambridge and Guy's man, Dr. Shufflebotham has for some time devoted himself to public health matters, and, residing at Newcastle-under-Lyme, is medical referee under the Workmen's Compensation Act to the North Staffordshire District.

Dr. Glover Lyon, who has lately been investigating the ventilation system of the House of Commons, and gave evidence before the Special Committee there last week, has for many years been attached to the City of London Hospital for Chest Diseases at Victoria Park, where he is now senior physician. An old student of Cambridge University, where he was a "wrangler" in the 'seventies, Dr. Glover Lyon went on to St. Thomas's for his clinical work, and, taking the qualifications of M.D. Cantab., M.R.C.P. Lond., and M.R.C.S. Eng., soon interested himself in the problems of tuberculosis. He eventually became the author of a new system of ventilation, whilst his interest in insurance work led to his founding the Life Insurance Medical Officers' Association, of which he was at one time president.

Mr. Henry William Armit, M.R.C.S., L.R.C.P., who has been appointed editor of the *Medical Journal of Australia*, is well known both as a medical journalist and as a scientific investigator. A former student of "Bart's.," Mr. Armit has the gift of languages, a circumstance which assisted him as representative of the *British Medical Journal* at various Continental congresses. To many medical men his work is chiefly known through his able translations of Forel's "Hypnotism and Psycho-Therapy," von Schrötter's "Hygiene of the Lung," and other books.

The new lecturer on materia medica, pharmacology, and therapeutics at Sheffield University, Dr. A. E. Barnes, is an M.B. of London as well as of Sheffield, and is also a member of the Royal College of Physicians. Formerly a research scholar of the British Medical Association, Dr. Barnes also holds the important appointment of physician to the Royal Infirmary at Sheffield, and has lately been tutor in clinical medicine at the University.

